# Efficient Identification of HIV Serodiscordant Couples by Existing HIV Testing Programs in South Brazil

**DOI:** 10.1371/journal.pone.0142638

**Published:** 2015-11-12

**Authors:** Christopher D. Pilcher, Claudia Alquati Bisol, Machline Paim Paganella, Snigdha Vallabhaneni, Leonardo Rapone da Motta, Sergio Kakuta Kato, Rosa Dea Sperhacke, Esper G. Kallas, Frederick M. Hecht, Ricardo Sobhie Diaz

**Affiliations:** 1 Division of HIV, Infectious Diseases, and Global Medicine, Department of Medicine, University of California San Francisco, San Francisco, CA, United States of America; 2 Laboratório de Pesquisa em HIV/AIDS, Centro de Ciências Biológicas e da Saúde, Universidade do Caxias do Sul, Caxias do Sul, Brazil; 3 Division of Clinical Immunology and Allergy, School of Medicine, University of São Paulo, São Paulo, Brazil; 4 Retrovirlogy Lab, Paulista School of Medicine, Federal University of São Paulo, São Paulo, Brazil; University of Missouri-Kansas City, UNITED STATES

## Abstract

**Objective:**

To examine the feasibility of identifying HIV negative at risk individuals in HIV serodiscordant couples, during voluntary HIV testing in South Brazil.

**Methods:**

We surveyed HIV testers at 4 public testing sites in Rio Grande do Sul. We obtained information on risk behaviors and sexual partnerships. HIV testing and testing for recent infection were performed; HIV prevalence and risk behaviors were assessed among subjects who reported having a steady partner who was HIV positive (serodiscordant group) and compared with the general testing population.

**Results:**

Among 3100 patients, 490 (15.8%) reported being in a steady relationship with an HIV positive partner. New HIV infections were diagnosed in 23% of the serodiscordant group (vs. 13% in the general population, p = 0.01); among newly positive subjects, recent HIV infections were more frequent (23/86, 26.7%) among testers with positive partners than among the general testing group (52/334; 15.6%; p = 0.016). Less than half of the serodiscordant testers reported having used a condom during the last sexual intercourse with their HIV-positive partner. Participants with inconsistent condom use with steady partner were four times more likely to test positive for HIV compared to those who reported always using condoms with the steady partner (OR: 4.2; 95% CI: 2.3 to 7.5).

**Conclusion:**

It is highly feasible to identify large numbers of HIV susceptible individuals who are in HIV serodiscordant relationships in South Brazil testing sites. Condom use within HIV serodiscordant couples is low in this setting, suggesting urgent need for biomedical prevention strategies to reduce HIV transmission.

## Introduction

Recent randomized clinical trials have shown that antiretroviral therapy (ART) can be highly effective at reducing sexual HIV transmission in HIV serodiscordant couples, whether given to the infected partner for Treatment as Prevention (TasP) [1], or given to an HIV negative partner as Pre-Exposure Prophylaxis (PrEP) [2]. Some observers have questioned the feasibility of delivering these interventions to HIV negative members of stable serodiscordant couples. A recent review of published data on the prevalence of HIV serodiscordance in 14 African nations questioned the feasibility of PrEP for HIV prevention in couples in hyperendemic countries, on the basis that the proportion of HIV negative individuals who are in serodiscordant couples is very low. They reasoned that targeted HIV-negative interventions would yield minimal impact on transmission [3]. Understanding how and whether HIV uninfected individuals in serodiscordant partnerships can be identified in the real world is an issue that is highly relevant to the distribution of resources to implementing proven HIV prevention strategies.

Brazil has embraced a particularly progressive approach to HIV prevention which has included providing free access to antiretroviral therapy since 1996. While these efforts have led to stabilization or decline of HIV infections among men who have sex with men (MSM) in most regions of Brazil [4], the South region (encompassing the states of Rio Grande do Sul, Parana and Santa Catarina) has experienced a marked increase in heterosexually transmitted subtype C HIV infection [5]. This regional trend has raised new calls for improved prevention efforts addressing needs of both MSM and heterosexual populations in Brazil. In this study, we focused on whether HIV serodiscordant couples at need of enhanced prevention services might be accessed through existing HIV testing sites in South Brazil.

## Methods

### Study Population

AMPLIAR Project study 010 was designed to characterize the epidemiology of the HIV testing population in South Brazil. Adult clients who were presenting for HIV testing were recruited at four voluntary HIV counseling and testing (VCT) centers in the state of Rio Grande do Sul (RS) in South Brazil. The study was conducted between October 2006 and January 2008.

### Study participants and Data Collection

At the participating sites, consecutive testing clients >16 years and with no history of a previous positive HIV test were approached and recruited by VCT counseling staff. Male and female participants of all ethnic groups were included. After informed consent, trained counselors administered a 20 minute structured questionnaire eliciting information on demographics; prior study participation; HIV history including testing, treatment, and exposure categories; sexual behaviors including partners, practices, condom use and sex work; drug use, and medical history. Participants were asked to report on risk behavior in the previous 12 months. Any man who reported having sex with another man in the past 12 months was classified as MSM, regardless of self-described orientation. All participants received pretest counseling from trained test counselors before venipuncture for serologic and antigen testing. Participation was anonymous, but information was gathered to identify repeat testers.

### Services and Referrals

Participation was offered to clients at public VCT sites, where HIV testing and pre- and post-test counseling by trained, experienced, professional counselors are offered free of charge. These VCTs are all located adjacent to specialized HIV treatment centers, where clients who test HIV-positive are referred for free comprehensive medical care, including STI treatment and combination antiretroviral therapy. Psychological counseling services and social workers are available at both VCTs and treatment clinics.

### HIV Testing and Staging

HIV status was determined by a reference standard including: 1) a 4th generation (HIV antigen-antibody “combo”) immunoassay, with 2) confirmatory HIV antibody testing (performed if reactive on the combo assay) and/or 3) Versant HIV-1 RNA 3.0 Assay (bDNA), Siemens Healthcare Diagnostics, Tarrytown NY, pooled 1:20, performed if the specimen was non-reactive on the combo assay [6,7]. Follow-up individual HIV RNA testing or additional confirmatory antibody tests were performed as necessary to confirm HIV status for all clients. The Aware BED EIA HIV-1 Incidence Test (Calypte Biomedical, Portland, OR; ODn cutoff = 0.8) was used to assess the likelihood of these newly diagnosed individuals having recent infections (of <6–12 months’ duration [8]).

### Ethical considerations

Procedures for this study were reviewed and approved by the institutional ethics boards at the Universidade de Caxias do Sul and the Brazilian National Committe for Ethics in Research (CONEP), and by the Committee on Human Subjects Research at the University of California, San Francisco. Written consent was obtained for all study participants. For minors < 18 years of age, written consent was obtained from the legal guardian prior to study enrollment.

### Data analysis

Participants who stated that their steady partner was HIV-positive were defined as being in a serodiscordant relationship. We calculated descriptive statistics for continuous variables, and comparison of the mean age between groups was done using t-test. Analysis of categorical variables consisted of frequencies and cross-tabulations, with χ^2^ tests to assess the significance of the associations. P-values <0.05 were considered statistically significant. Univariate and multivariate logistic regression were used to determine odds ratios and 95% confidence intervals. All statistical analyses were performed using Stata software version 11.0 (College station, Texas).

## Results

### Demographic characteristics

We enrolled a total of 3590 participants seeking HIV testing at participating counseling and testing sites. Four hundred and ninety test seekers reported negative or unknown HIV status and also reported that they were in a steady relationship with an HIV infected partner: these were categorized as serodiscordant testers. The remaining 3100 were considered general HIV-test seekers. As seen in Table 1, approximately half of participants in both groups were male, and more than two-thirds in each group self-identified as being White. Participants in the serodiscordant group were slightly older with mean age of 36.2 (standard deviation (SD):10.5) compared to mean age of 33.9 (SD:12.0) among the general HIV testing population. Significantly more participants in the serodiscordant group had less than an 8^th^ grade education (46.9%) compared to general HIV testers (42.1%, p = 0.04). Sexual orientation of participants was similarly distributed among both groups with 6–7% of each group comprised of MSM.

**Table 1:** Demographic characteristics of the entire cohort and risk factors for HIV infection.

Characteristic	Non-serodiscordant participants	Serodiscordant participants	P values
	N(%)	N(%)	
**All Participants**	3100 (100%)	490 (100%)	n/a
**Male gender**	1588 (51.2)	269 (54.9)	0.13
**Mean age**	33.9 (SD:12.0)	36.2 (10.5)	0.0001
**Race**			0.67
White	2259 (72.9)	352 (71.8)	
Black	451 (14.6)	68 (13.9)	
Mixed	358 (11.5)	65 (13.2)	
**Urban residence**	2699 (87.5)	397 (81.3)	0.002
**Education**			
<8 yrs school	1304 (42.1)	230 (46.9)	0.04
**Sexual Orientation**			0.32
MSM	197 (6.4)	35 (7.1)	
Heterosexual males	1392(44.9)	234 (47.7)	
All women	1511 (48.7)	221(45.1)	
**HIV positive**	413(13.3)	113 (23.1)	<0.01

### Sexual risk behavior of participants in serodiscordant relationships

Number of partners and condom use behavior of participants in serodiscordant relationships are summarized in Table 2, and are separated by heterosexual male (n = 234), MSM (n = 35), and female participants (n = 221). Median time since the last HIV test was 11 months (IQR: 4–23). Seventy percent of heterosexual men, 45.7% of MSM, and 86.9% of women reported having only one sexual partner in the last 12 months. Less than half of participants reported using a condom at last sex intercourse with their steady, HIV-positive partner, and only one quarter of heterosexual men to one third of MSM and women reported using condoms consistently in the last twelve months with this partner. The most common reasons stated for not using condoms regularly with their steady partner was Trust in my partner (“Confia no parceiro”) (n = 107/338; 31.7%), followed by Don’t like condoms (“Não gosta de usar camisinha”) (51/338; 15.1%), and Partner doesn’t accept (“Parceiro não aceita”) (30/338; 8.9%), consistent with barriers to condom use in discordant couples that have been previously reported in Brazil [9,10]. Those who did not consistently use a condom with their steady sex partner were at four times greater odds of testing HIV positive compared to those who reported always using a condom with the steady partner (OR: 4.2; 95% CI: 2.3–7.5). Condom use with a casual partner was somewhat higher, with 58.5% (heterosexual men) to 75% (MSM and women) reporting condom use at last sex with a casual partner. Overall, ten percent (n = 49) had been diagnosed with an STI in the last 12 months, with rates slightly higher among MSM (14.3%) and women (10.9%) compared to heterosexual men (8.5%).

**Table 2:** Sexual risk behavior of the 490 participants in serodiscordant partnerships.

Variable name	Heterosexual Men	MSM	Women
	N = 234	N = 35	N = 221
Number of sex partners in the last 12 months			
1	164 (70.0%)	16 (45.7%)	192 (86.9%)
2–4	50 (21.4%)	16 (45.7%)	21 (9.5%)
>5	17 (7.3)	3 (8.6%)	5 (2.3%)
Condom use with steady partner in the last 12 months			
Always	60 (25.6%)	11 (31.4%)	81(36.7%)
Sometimes	85 (36.3%)	14 (40%)	70 (31.6%)
Never	86(36.8%)	10 (28.6%)	69 (31.2%)
Condom use at last sex with steady partners	102 (43.6%)	17 (48.6%)	109 (49.3%)
Condom use with casual partner in the last 12 months			
Always	39/65 (60%)	14/20 (70.0%)	17/28 (60.7%)
Sometimes	7/65 (10.8%)	3/20 (15.0%)	4/28 (14.3%)
Never	19/65 (26.2%)	3/20 (15.0%)	7/28 (25%)
Condom use at last sex with casual partner	38/65 (58.5%)	15/20 (75%)	21/28 (75%)
Diagnosed with an STI in the last 12 months	20 (8.5%)	5 (14.3%)	24 (10.9%)

### HIV testing history

Prevalence of HIV infection was much higher in the serodiscordant tester group than the general testing population (23.1% versus 13.3%, p<0.001). Those in serodiscordant relationships had almost twice the odds of being HIV positive than the general testing population (OR: 1.8; 95% CI:1.4–2.3) after controlling for age, gender, race, education, and sexual orientation.

Staging information was available on 420 (80%) of the 526 HIV-infected persons in the entire cohort (334/413 (81%) in the general testing population and 86/113 (76%) in the serodiscordant group). A significantly greater proportion of HIV-positive persons in the serodiscordant group had serology suggesting recent HIV infection (23/86; 26.7%) compared to the general testing group (52/334; 15.6%) (p = 0.016). When asked about why they were seeking testing, most individuals in serodiscordant partnerships (309/487, 63.5%) responded that they “had been exposed to a risky situation”.

## Discussion

In a region-wide sample involving 3,590 HIV test-seekers in South Brazil, we found that a striking proportion (14%) of testers reported, when asked, that they were in serodiscordant relationships with an HIV positive individual. We found that these individuals were indeed at high risk for HIV infection: their odds of testing HIV positive were nearly twice that of the general population seeking HIV testing, and their odds of having acute or recent infection were nearly four times as high as other testers, even when controlling for other risk factors for HIV acquisition. These data confirm that members of HIV, serodiscordant partnerships with ongoing, elevated risk of HIV acquisition can be readily identified in South Brazil, using the existing HIV testing infrastructure. The degree of risk seen in this group demonstrates a need for targeted HIV prevention services.

It has been recently argued [3] that biomedical HIV prevention efforts for serodiscordant couples should be targeted or advertised primarily to HIV positive partners, because members of serodiscordant couples will be too rare among the general HIV seronegative population to be easily identified by existing programs. This was not the case in South Brazil, where in the process of identifying 413 new HIV infections, an existing testing system identified HIV 377 additional individuals who were in a serodiscordant couple. In this population, simply asking seronegative test-seekers if they had a steady HIV positive partner effectively doubled the number of individuals who could be specifically targeted for biomedical interventions proven to reduce HIV transmission; if services could be provided for their HIV positive partners the potential impact of this strategy would be even greater.

The elevated risk of HIV infection for individuals in serodiscordant couples is explained in part by low rates of condom use within these steady partnerships. For the minority of the HIV testers who reported always using condoms with their positive partner, rates of infection were reduced by four-fold. However, key barriers to condom use cited by the seronegative partners in this study included refusal of the HIV positive partner to use condoms, and also trust in the HIV positive partner.

The presence of these barriers to consistent condom use in such a large group of people at very high risk of HIV transmission suggests a major need and opportunity to target the use of other proven behavioral and biomedical interventions such as treatment as prevention (TasP) [1], pre-exposure prophylaxis (PREP) [2], and male medical circumcision (MMC) [11]. Each of these interventions has been proven to be effective at preventing HIV transmission in discordant couples when added to condom use and counseling. Additional interventions directed at couples (e.g., Couples HIV Testing and Counseling, CHTC) are one way to gain buy-in from the HIV-positive partner and can result in increased condom use in discordant couples [12]. Improving access of serodiscordant partners to both biomedical and behavioral interventions could be important to reducing the transmission of HIV infection in discordant couples.

These data are from a small number of HIV counseling and testing sites in three cities in Rio Grande do Sul state, Brazil, and may not be generalizable to testing sites in other areas of the world. The epidemic in South Brazil is similar in many ways to epidemics in sub-Saharan Africa, with relatively high rates of infection among heterosexuals and subtype C as the most prevalent HIV subtype [5]. Testing sites serving IDU or MSM risk communities with higher partnership rates might expect to encounter lower numbers of individuals in steady serodiscordant partnerships (although we found similar proportions of MSM testers and non-MSM testers that reported having HIV positive steady partners). In this study of HIV testers, we were unable to assess whether their HIV positive partners might be on (or adherent to) antiretroviral therapy already. The ability to obtain this information, as well as information on potential concurrent relationships, would enhance the delivery of appropriate interventions. Comprehensively assessing such couples for transmission risk remains a key challenge for implementing proven prevention strategies and biomedical interventions.

In the new prevention landscape, HIV testing sites need to become sites at which individuals screening for HIV infection are also assessed for membership in an HIV serodiscordant partnership. The Brazilian national guidelines for health providers emphasize the importance of partner disclosure as well as HIV testing for partners of patients who are newly-diagnosed [13]. Since the time this study was completed, the Department of STD, Aids and Viral Hepatitis (part of Brazilian Ministry of Health's Surveillance Secretariat) has continued to shape its public health strategies for prevention of HIV transmission, and has recently joined a handful of other countries in providing free, government sponsored antiretroviral TasP for HIV positive individuals who are in discordant couples regardless of CD4 cell count [14]. Additionally, there is an ongoing implementation project across multiple sites in Brazil to assess acceptance, feasibility and optimal methods for delivering PrEP as HIV prevention [15]. If the world is to move toward an AIDS-free generation [16], existing programs for HIV testing have a key role to play in implementation of new biomedical prevention strategies.
